# A Blueprint for Clinical-Driven Medical Device Development: The Feverkidstool Application to Identify Children With Serious Bacterial Infection

**DOI:** 10.1016/j.mcpdig.2024.10.003

**Published:** 2024-10-30

**Authors:** Evelien B. van Kempen, Sanne E.W. Vrijlandt, Kelly van der Geest, Sophie Lotgering, Tom A. Hueting, Rianne Oostenbrink

**Affiliations:** a Department of Paediatrics, Juliana Children’s Hospital Haga Hospital, Den Haag, the Netherlands; b Department of Paediatrics, Erasmus MC Sophia Children’s Hospital, Rotterdam, the Netherlands; c Department of Medical Technology, Erasmus MC, Rotterdam, the Netherlands; d Department of Information & Technology, Erasmus MC, Rotterdam, the Netherlands; e Evidencio, Haaksbergen, the Netherlands

## Abstract

Clinical decision rules (CDRs) integrated into applications enhance diagnostic and treatment prediction support for clinicians, necessitating Confirmité Europeenne (CE)-mark certification to enter the European market. We describe the development of a CDR as a medical device, focusing on challenges from a physician’s perspective exemplified by the Feverkidstool (FKT), a validated CDR for febrile children. We pursued a local process, aligned with the CE-marking process, to develop the FKT as in-house developed device. We aimed to provide a blueprint for colleagues. Medical device development, conforming the medical device regulation and performed by a multidisciplinary team, encompassed 5 stages: market scan, design, production, verification and validation and conformity assessment. Regulatory processes were continuously updated. The market scan identified a need for the FKT compared with existing applications. A prototype was designed in stage 2, further adjusted and improved based on the qualitative and quantitative results of stages 2-4. Lastly, stage 5 confirmed FKT’s performance and safety. Medical device development presents challenges for physicians, requiring collaboration for technical, regulatory, and financial expertise. Multidisciplinary teamwork also poses challenges, including uncertainties regarding responsibility and timelines. After CE certification, adapting to evolving needs and ensuring data privacy highlights the ongoing nature of medical device development.

Software applications incorporating clinical decision rules (CDRs) for prediction, diagnoses, and treatment are considered medical devices and have to be in adherence with the Medical Device Regulations (MDR) in Europe (Supplemental Table 1, available online at https://www.mcpdigitalhealth.org/).^1^ As most clinical decision tools (CDTs) have a diagnostic or therapeutical purpose, they generally classify as a medical device class IIa or higher. Those have to undergo a conformity assessment procedure by a notified body (independent authority), before affixation of the Confirmité Europeenne (CE) mark can occur to allow placement on the European Union market, confirming product adherence with the MDR (EU) 2017/745 and meeting the General Safety and Performance Requirements (GSPRs).^1^^,^^2^

Medical device development consists of 5 stages, combined with continuous regulatory processes depicted in Figure for our setting.^3^^,^^4^ Challenges concern among others clinical, technical, and regulatory aspects.^5^ To assist clinicians, the Erasmus Medical Center (EMC) has established a local process to develop medical devices in-house, also serving as base for obtaining CE-marking. We followed this process to develop the Feverkidstool (FKT) CDR as application to enable its wide clinical implementation.

The FKT is a validated CDR to standardize risk assessment of serious bacterial infections (SBIs) and support clinical decision making in febrile children between 1 month and 18 years.^6^^,^^7^ The methodologic development of CDRs include 3 stages: derivation, validation (narrow and broad), and impact analysis, each stage supported by scientific evidence.^8^^,^^9^ The most valuable evidence for a CDR lies in extensive validation and found impact, indicating the reliability of a CDR’s applicability across diverse settings and enhance patient outcomes.^8^^,^^9^ Although several CDRs for identifying children with SBI exist, only few are validated, fewer underwent impact analysis, and they are rarely used in clinical practice.^10^, ^11^, ^12^, ^13^, ^14^, ^15^ As the FKT has proven diagnostic accuracy, been crossvalidated and externally validated, and found impact, it is suitable for widespread implementation in pediatric emergency care.^6^^,^^7^^,^^16^ The envisioned product was a web-based application, accessible by mobile phone and computer, providing a risk score based on entered patient characteristics . This article reviews this process and aims to provide a blueprint to guide colleagues.

### Methods

The project ran between February 2022 and December 2023 at Erasmus Medical Center Sophia Hospital, Netherlands. The 5 stages of medical device development were pursued in adherence with the MDR: market scan and justification, design, production, verification and validation, and conformity assessment. Regulatory processes continued throughout and were continuously updated. This led to a technical dossier on efficacy, safety, and risks. Approval from the Medical Ethics Research Committee Erasmus Medical Center was obtained (MEC-2022-0221).

#### Working Group

The working group consisted of clinicians with expertise in pediatric emergency medicine, clinicians with expertise in epidemiology of EMC, medical technology experts of EMC, Quality Assurance and Regulatory Affairs office of EMC, privacy officers of EMC, and the manufacturer. The core group consisted of the authors (E.B.v.K., K.v.d.G., T.A.H., and R.O.) and those from the EMC application development group (S.L. and K.C.). The review group were Dutch pediatricians with an expertise in pediatric emergency medicine (J.B., M.I.v.V., J.P., A.L., and D.H.F.G.) and residents in pediatrics (J.Z., S.v.d.B., W.v.H., E.E., and K.d.R.). The clinical pediatric emergency experts and the review group were approached via the national network on pediatric emergency medicine and selected on availability. Participation was based on voluntarily participation.

#### Regulatory Processes

According to the classification rules in Annex VIII of the MDR, the FKT application was considered a class IIa medical device. A dossier was established, complying to MDR art 5.5, to allow for in-house production and usage without intervention from a notified body. This saved time in the certification process. We chose a software supplier with the experience, proposition, and technical platform to handle the regulatory process, licensing, and distribution. The following definitions were adhered:

- Intended purpose: FKT advises on more targeted antibiotic prescription in children with fever presenting at the emergency department (ED), leading to less antibiotic prescriptions in children who do not profit from antibiotic treatment and less therapeutic failure in children who benefit from antibiotic treatment;
- Intended users: pediatricians and pediatric residents, working at the ED;
- Intended population: FKT is validated for febrile children aged 0 to 18 years presenting at the ED.

Technical documentation demonstrating the FKT meets the GSPRs was gathered accordingly (Supplemental Table 2, available online at https://www.mcpdigitalhealth.org/). A risk management file and matrix was developed and updated during each stage, with different hypothesized scenarios with their encountered risks, and classified based on severity, risk of occurrence, and probability of intervention. Mitigation measures were proposed to counteract potential risks and mainly included disclaimers for the user and additional information on the purpose of the application and target population (Supplemental Table 3, available online at https://www.mcpdigitalhealth.org/). Regulatory documents from stage 1 were regularly updated throughout the other phases. Risks and performance will be continued to be evaluated during postmarketing surveillance.

### Stage 1: Market Scan and Justification

A market scan of current existing applications is required to justify the need for the application to be developed. The aim is to determine the current state-of-the-art and to evaluate if no equivalent application meeting the essential requirements exists. As not all devices, in particular commercial solutions, are published in scientific journals, it is important to expand the search beyond scientific resources to so-called gray literature and, for example, Google. For the FKT, we performed a market scan on tools available on software applications for children between 0 and 18 years presenting with fever at the ED (Supplemental Figure 1, available online at https://www.mcpdigitalhealth.org/). The target user group included pediatricians and pediatric residents. An initial Google and PubMed search resulted in 11,000,000 sites and 25 articles, respectively, from inception until November 2023. The Google search was limited to the first 5 pages (47 sites). After screening, 5 comparable applications were included: Step-by-Step, PedsGuide, ALgorithms for the MANagement of Acute CHildhood illnesses, ePOCT, and Pediatric Emergency Care Applied Research Network rule (Table 1).^10^, ^11^, ^12^, ^13^^,^^17^ Unlike the FKT, none were intended for children older than 5 years, and most were consensus based without an impact analysis to prove effectiveness. Therefore, we deemed that the market scan found a need and justification for the FKT application.

**Table 1:** Overview of the Identified Applications vs Wanted Requirements

Requirements	Step-by-Step	PedsGuide	ALMANACH	ePOCT	PECARN rule
Age, 0-18	No, 0-90 d	No, 0-90 d	No, 2 mo to 5 y	No, 2 mo to 5 y	No, <60 d
Level of CPR	Validated clinical prediction rule	Consensus based clinical algorithm	Clinical algorithm as decision support	Clinical algorithm as decision support including point of-care tests	Clinical algorithm as decision support including point of-care tests
Free accessible	Yes	Yes	Only on Android for the participating hospitals	Only on Android for the participating hospitals	Yes
CE certification or FDA approval	No	No	No	No	No

ALMANACH, ALgorithms for the MANagement of Acute CHildhood illnesses; CE, Confirmité Europeenne; CPR, clinical prediction rule; FDA, Food and Drug Administration; PECARN, pediatric emergency care applied research network.

### Stage 2: Design

We developed the software in a private-public partnership. Our manufacturer developed a prototype of the FKT application together with members of the core group. This warranted to obtain end-user input in an early phase, by either qualitative or quantitative methods.^18^ Qualitative methods provide rich in-depth information and allow for exploring important topics like facilitators, barriers, feasibility, acceptability, and improvements in layout.

In 10 semistructured interviews and 1 workshop with pediatricians and pediatric residents working with the first FKT application prototype, we identified user requirements. Questions were derived from a.o. Ottawa Acceptability of Decision Rules Instrument and the System Usability Scale.^19^^,^^20^ In addition, topics concerning user interface and incorporation into clinical workflow were discussed. Thematic analysis by Braun and Clarke^21^ identified 4 themes from the semistructured interviews: clinical decision support, feasibility of the FKT application prototype, factors for successful implementation, and MDRs (Table 2). On the basis of these results, adjustments to the prototype were made (Table 2). The improved FKT prototype was next rated positively during the workshop. Further suggested improvements for the final prototype (Supplemental Figure 2, available online at https://www.mcpdigitalhealth.org/) concerned change in sequence of variables according to electronic health record, automatic data saving, clear display of value ranges, and warning on values under or above the accepted range. To ensure security, 2-factor authentication was realized. The final prototype shown in Supplemental Figure 2.

**Table 2:** Overview Themes and Subcategories Identified During Semistructured Interviews and Workshops

Theme	Category	Main result	Adjustments
Clinical decision support	Attitude toward clinical decision support	• Most participants do not regularly use clinical decision support • Participants would not make decisions solely based on the advice of the FKT	
	FKT as support for clinical decision making	• Reasons to use FKT are as follows: - support in prescribing antibiotics - support in addressing antibiotic resistance and validating clinician’s treatment plan toward parents • Participants would not use the FKT application in case of clear diagnosis, because of no added value • 3 pediatricians: FKT device would be more useful for less experienced clinicians, experienced pediatricians do not necessarily need extra decision support	
Feasibility of the FKT application	Prototype is user-friendly	• FKT application prototype was easy to use, quick to fill in, and available as wished for on both smartphones and computers	
	Adjustments for improvement of user-friendliness	• Following suggestions were made: automated data entry presentation of FKT percentage score as risk group and ensuing advice on whether to prescribe antibiotics, display of abnormal values of variables, a more inviting layout, usage of 1 language instead of Dutch and English interchangeably, and removing unnecessary features	• Automated current date • Risk meter for the calculated risk including an advice for antibiotic prescription per risk group • Display of abnormal values of variables • More inviting layout • Pictograms • All text in Dutch
Factors for successful implementation	Barriers for regular usage of the FKT	• Following barriers were mentioned: lack of time, usage of FKT easy to forget in a busy emergency department, not always known CRP level (yet part of the prediction rule), and no clear role in more complex patients with comorbidities to whom antibiotics are more easily provided. Earlier mentioned improvements like integration in the EHR could improve this barrier	
	Requirements for successful implementation	• Directly available and easy to find • Awareness on availability • User experience to acknowledge usefulness	
Medical device regulations		• Visibility of the scientific background • Disclaimer on intended use (patient population), legal protection and privacy/data protection and storage	• Information about the scientific background • Disclaimer

Main results on which themes and subcategories are based are displayed. The adjustments based on these results made are shown.

CRP, C-reactive protein; EHR, electronic health record; FKT, Feverkidstool.

### Stage 3: Production

The algorithm used in the application requires validation against the original one, as clinical definitions or cutoff points being used in the (validated) algorithm may have changed over time, resulting in a different categorization. We considered the highest possible risk for errors to occur with variables having values around the cutoff points for critical parameters in the CDR.

We validated the algorithm used in the FKT prototype against the original algorithm through SPSS version 25, by comparing scores using data from 95 cases with values around the cutoff points for the critical parameters from an existing database of febrile children.^6^ Mismatch of scores using the original algorithm vs FKT prototype occurred in 10 of the 95 scores, all due to different definitions of abnormal vital signs. These differences arose from using different editions of advanced paediatric life support guidelines in time and were resolved by adjusting definitions to the same edition.

### Stage 4: Verification and Validation

Verification determines whether the product functions as it should work according to the requirements and includes both stability of statistical model as stability of interface and software performance (general usability). Validation determines whether the product complies with the requirements of the user by evaluating user experience (quality assurance). We asked 13 future users to complete the FKT for 7 to 10 cases to assess the accuracy of data entry, for example, correct data entry and the occurrence of typos. We confirmed the concordance between the data entered into the FKT application and the same patient’s data in their electronic health record. These users also completed self-created surveys regarding visual representation, technical elements, duration, evaluation of previous adjustments, and other aspects of user-friendliness after entering patient cases. They were satisfied regarding visual representation, technical aspects, acceptance, and user-friendliness. The FKT was completed on average within 1 minute 52 seconds, deemed feasible for the ED.

To further improve the prototype, suggestions were put forward and subsequently realized: (1) providing a risk score for when not all variables are known; (2) improving the calendar function and (3) giving clear instructions on what numbers to enter. These results found that usability met the previous established demands.

### Stage 5: Conformity Assessment

Lastly, data gathered during the continuous clinical evaluation should verify the safety and performance, demonstrating that its performance outweighs potential risks. The product needs to meet the GSPR and the clinical benefits claimed by the manufacturer. We predefined the claims on performance, safety, and intended use of the CDT (Supplemental Table 4, available online at https://www.mcpdigitalhealth.org/). Regarding performance, all predefined claims were supported by data from earlier studies (Prins EAB, Oostenbrink R. Master thesis: Feverkidstool: from medical decision tool to certified medical device. Department of General Paediatrics, Erasmus MC Sophia Children’s Hospital, Rotterdam, Netherlands, 2022).^6^^,^^7^^,^^16^^,^^22^ The FKT running on manufacturer developed software preserved the required (technical) performance. For safety, we identified 2 risks from the available data sets. First, the FKT uses a 10% threshold for high-risk groups and ensuing advice to start antibiotic treatment. So, for those with a risk below this threshold, up to 10 of the 100 patients would have a SBI for which treatment would likely be beneficial. This is considered to be acceptable, as a minimum of 90 of the 100 patients would not benefit from antibiotic treatment plus the same risk is faced using state-of-the-art guidelines. Second, no FKT score can be calculated without C-reactive protein level. Consequently, an adjustment has been made providing a risk score range when C-reactive protein is not entered. The risk/benefit profile of the FKT application was concluded positive.

### Submission and Commissioning of the CDT

The EMC Quality Assurance and Regulatory Affairs has approved the documentation. A product dossier was required by the Dutch Health and Youth Care Inspectorate, to ensure adherence with requisites for procurement and safe application. As the FKT incurred no additional costs, this process was completed with approval of documentation formulated in the aforementioned stages. The FKT was submitted to a notified body to review all documentation and obtained CE-mark (October 2024; available on feverkids.evidencio.com).

### Discussion

Using the FKT as model, we provided a blueprint to perform the medical device development process. Our application development benefited from multidisciplinary teamwork. Our in-house certification process fastened the process for in-house use compared with that governed by MDR regulations (involving a legal manufacturer). We found consistent functionality, mitigated risks, and satisfactory usability. The benefit, measured in terms of performance tested against predefined claims, outweighed potential risks managed by feasible mitigation measures.

#### Interdisciplinary Collaboration

Collaboration with different experts is important to support clinicians through the medical device development process. Previously identified barriers include limited understanding of the regulatory requirements by clinicians, lack of funding, and changing environment of information technology and security.^23^ The various stakeholders (depicted in Figure) are prerequisites to successfully execute the proposed approach and to allow for generalizability. We experienced both the strengths and challenges of interdisciplinary teamwork. In-house development of medical devices is a rather new phenomenon, with the FKT application being the second at the EMC. Locally established guidance and expertise and checklists provided by the medical technology department facilitated documentation adherence with MDR requirements. Technical expertise of our private-public partner facilitated the CDT’s design and development and provision of information for technical documentation. Learning points were the need for more clarity regarding the essential documents for the in-house process, defined responsibilities and timelines. Developing a strategy including these factors could streamline the process.

**Figure:**
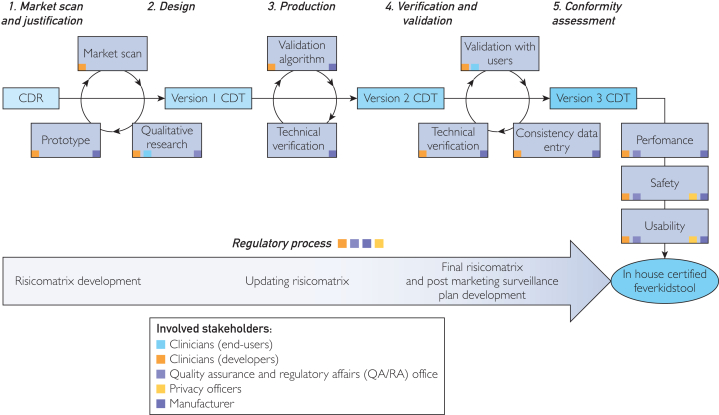
Overview of the 5 development phases according to medical device development. Within each phase, the steps that should be performed are depicted. The different stakeholders involved per phase and steps are shown. It is important to recognize it involves an iterative process. CDR, clinical decision rule; CDT, clinical decision tool.

#### Tasks Associated With Commissioning the Device

In-house certification contributes to local implementation, although CE certification facilitates wide distribution. Because passive implementation of CDTs has been shown to be insufficient, we have developed an implementation strategy using the consolidated framework for implementation research framework.^24^ Involving key stakeholders is important. After national implementation, broad adoption of the tool necessitates expanding collaboration from international scene. Clinical performance, risks, and implementation outcomes will be investigated through postmarket surveillance and clinical follow-up performance, which are mandatory facets of the CE certification process.^1^ This will also entail evaluating if similar devices are created, expanded with a patent search. Moreover, acquiring a postmarket surveillance plan before introducing a tool does not account for necessary scientific adjustments to a CDT after implementation. Over time, epidemiologic changes or new biomarkers may necessitate updates to the tool, requiring a different evaluation approach for the FKT. This responsibility lies with the tool’s developer and extends beyond the CE certification. Additionally, software disruptions and off-label use may pose challenges when scaling up. These considerations highlight that the implementation process for a CDT continues beyond obtaining a CE-mark.

### Conclusion

We found the 5-stage approach plus regulatory process of medical device development, with the FKT application as an example. Although development of CDR is scientifically oriented and feasible with statistical knowledge, development of medical devices requires collaboration to have the technical, regulatory, and financial expertise needed. Involving numerous stakeholders with different expertise underscores the necessity to work as a team. Although the available guideline documents proved advantageous in addressing associated challenges with teamwork, enhancements in the process could be achieved by specifying the individual ultimately responsible for a given phase and implementing predefined deadlines.

## Potential Competing Interests

Dr Hueting is employed by Evidencio; Evidencio was paid for developing the Feverkidstool application and for writing technical documentation. Dr Oostenbrink reports support from Dutch National Health Council (ZonMW), funding paid to her institution, 2021-2023. The other authors report no competing interests.

## References

[1] Regulation (EU) 2017/745 of the European Parliament and of the Council of 5 April 2017 on medical devices, amending Directive 2001/83/EC, Regulation (EC) No 178/2002 and Regulation (EC) No 1223/2009 and repealing Council Directives 90/385/EEC and 93/42/EEC (Text with EEA relevance.) European Union. https://eur-lex.europa.eu/legal-content/EN/TXT/?uri=CELEX%3A32017R0745.

[2] (2023). Extension of the MDR transitional period and removal of the ‘sell off’ periods.

[3] MDR. Medische hulpmiddelen ontwikkeling en vervaardiging. Rotterdam: Erasmus MC. https://kms.erasmusmc.nl/iDocument/Viewers/Frameworks/ViewDocument.aspx?documentid=fc955c25-aead-4149-a883-66cb0a9c8543&customcss=&HyperlinkID=5a5422c6-16c9-4778-9582-e3f09723ca7b.

[4] Aune A., Vartdal G., Jimenez Diaz G., Gierman L.M., Bergseng H., Darj E. (2023). Iterative development, validation, and certification of a smartphone system to assess neonatal jaundice: development and usability study. JMIR Pediatr Parent.

[5] Espinoza J., Shah P., Nagendra G., Bar-Cohen Y., Richmond F. (2022). Pediatric medical device development and regulation: current state, barriers, and opportunities. Pediatrics.

[6] Nijman R.G., Vergouwe Y., Thompson M. (2013). Clinical prediction model to aid emergency doctors managing febrile children at risk of serious bacterial infections: diagnostic study. BMJ.

[7] van de Maat J.S., Peeters D., Nieboer D. (2020). Evaluation of a clinical decision rule to guide antibiotic prescription in children with suspected lower respiratory tract infection in the Netherlands: a stepped-wedge cluster randomised trial. PLoS Med.

[8] Reilly B.M., Evans A.T. (2006). Translating clinical research into clinical practice: impact of using prediction rules to make decisions. Ann Intern Med.

[9] McGinn T.G., Guyatt G.H., Wyer P.C., Naylor C.D., Stiell I.G., Richardson W.S. (2000). Users’ guides to the medical literature: XXII: how to use articles about clinical decision rules. Evidence-Based Medicine Working Group. JAMA.

[10] Pantell R.H., Roberts K.B., Adams W.G. (2021). Evaluation and management of well-appearing febrile infants 8 to 60 days old. Pediatrics.

[11] Gomez B., Mintegi S., Bressan S. (2016). Validation of the “step-by-step” approach in the management of young febrile infants. Pediatrics.

[12] Keitel K., Kagoro F., Samaka J. (2017). A novel electronic algorithm using host biomarker point-of-care tests for the management of febrile illnesses in Tanzanian children (e-POCT): a randomized, controlled non-inferiority trial. PLoS Med.

[13] Rambaud-Althaus C., Shao A.F., Kahama-Maro J., Genton B., d’Acremont V. (2015). Managing the sick child in the era of declining malaria transmission: development of ALMANACH, an electronic algorithm for appropriate use of antimicrobials. PLoS One.

[14] Cowley L.E., Farewell D.M., Maguire S., Kemp A.M. (2019). Methodological standards for the development and evaluation of clinical prediction rules: a review of the literature. Diagn Progn Res.

[15] Kingma A.E.C., van Stel H.F., Oudega R., Moons K.G.M., Geersing G.J. (2017). Multi-faceted implementation strategy to increase use of a clinical guideline for the diagnosis of deep venous thrombosis in primary care. Fam Pract.

[16] de Vos-Kerkhof E., Nijman R.G., Vergouwe Y. (2015). Impact of a clinical decision model for febrile children at risk for serious bacterial infections at the emergency department: a randomized controlled trial. PLoS One.

[17] McCulloh R.J., Fouquet S.D., Herigon J. (2018). Development and implementation of a mobile device-based pediatric electronic decision support tool as part of a national practice standardization project. J Am Med Inform Assoc.

[18] Money A.G., Barnett J., Kuljis J., Craven M.P., Martin J.L., Young T. (2011). The role of the user within the medical device design and development process: medical device manufacturers’ perspectives. BMC Med Inform Decis Mak.

[19] Brehaut J.C., Graham I.D., Wood T.J. (2010). Measuring acceptability of clinical decision rules: validation of the Ottawa acceptability of decision rules instrument (OADRI) in four countries. Med Decis Making.

[20] Brooke J., Jordan P.W., Thomas B., McClelland I.L., Weerdmeester B. (June 1996). Usability Evaluation in Industry, Chapter 21.

[21] Braun V., Clarke V. (2014). What can “thematic analysis” offer health and wellbeing researchers?. Int J Qual Stud Health Well-being.

[22] Hagedoorn N.N., Wagenaar J.H.L., Nieboer D. (2021). Impact of a clinical decision rule on antibiotic prescription for children with suspected lower respiratory tract infections presenting to European emergency departments: a simulation study based on routine data. J Antimicrob Chemother.

[23] Rose L., McCaney J., Dave G. (2023). Software to manage regulatory workflows for medical device development at academic medical centers: a critical gap. J Clin Transl Sci.

[24] Damschroder L.J., Aron D.C., Keith R.E., Kirsh S.R., Alexander J.A., Lowery J.C. (2009). Fostering implementation of health services research findings into practice: a consolidated framework for advancing implementation science. Implement Sci.

