# Supplementary material for: A Blueprint for Clinical-Driven Medical Device Development: The Feverkidstool Application to Identify Children With Serious Bacterial Infection
Source: Mayo Clin Proc Digit Health. 2024 Oct 30;2(4):656–64. doi: 10.1016/j.mcpdig.2024.10.003 (PMC11975846; doi:10.1016/j.mcpdig.2024.10.003)

Supplementary Material

Supplementary Table 1 page 2

Supplementary Table 2 page 3

Supplementary Table 3 page 4

Supplementary Figure 1 page 5

Supplementary Figure 2 page 6

***Supplementary Table 1**: definition medical device by EU Regulation on Medical Devices (1). In the USA, the FDA's Center for Devices and Radiological Health and in Australia the Therapeutic Goods Administration are in place*

**Supplementary Table 1: Definition medical device by EU Regulation on Medical Devices**
‘medical device’ means any instrument, apparatus, appliance, software, implant, reagent, material or other article intended by the manufacturer to be used, alone or in combination, for human beings for one or more of the following specific medical purposes:<br>— diagnosis, prevention, monitoring, prediction, prognosis, treatment or alleviation of disease,<br>— diagnosis, monitoring, treatment, alleviation of, or compensation for, an injury or disability,<br>— investigation, replacement or modification of the anatomy or of a physiological or pathological process or state,<br>— providing information by means of *in vitro* examination of specimens derived from the human body, including organ, blood and tissue donations,<br><br>and which does not achieve its principal intended action by pharmacological, immunological or metabolic means, in or on the human body, but which may be assisted in its function by such means.<br><br>The following products shall also be deemed to be medical devices:<br>— devices for the control or support of conception;<br>— products specifically intended for the cleaning, disinfection or sterilisation of devices as referred to in Article 1(4) and of those referred to in the first paragraph of this point.

***Supplementary Table 2**: overview subsets technical documentation with used standards*

**Subsets technical documentation**	**Applied standards**
Software development	IEC62304
Risk management	ISO14971
Quality management	ISO13485
Information security	ISO27001 and NEN7510
Usability engineering	IEC62366
Clinical evaluation	MDR art. 61 and Annex XIV and MEDDEV 2.1/7 rev 4

Supplementary Table 3: a simplified version of the risk matrix for main risks during use of the FKT application

**Risk**	**Event**	**Situation**	**Possible harm**	**Mitigation strategy**	**Results in phase 5**
User is no physician in hospital setting	Tool is not validated for usage in primary care nor for other users than pediatricians	Over- or underestimation of risk	Side effects of antibiotics or ongoing infection	Disclaimer in application	In this setting only physicians working at the Emergency Department treating children could access the webbased application using a two step authentication. This will be evaluated post-marketing phase
Algorithm contains mistake	Tool predicts higher risk than actually is<br><br>Tool predicts lower risk than actually is	Overusage of antibiotics<br><br>Deterioration of health condition	Side effects of antibiotics<br><br>Ongoing infection	Disclaimer stating: only professional usage by physician, physician is responsible, tool is supportive	During technical validation and verification and prior testing by developer, this did not occur. No errors occured. Yet, this will be evaluated during PMCF
Tool is not functioning	Tool gives error	Working according to (previous) standard of care	Not applicable	Service Level Agreement	During technical validation and verification, this did not occur. This will be evaluated during PMCF
User enters values in wrong entity	Tool produces wrong outcome	Over- or underestimation of risk	Side effects of antibiotics<br><br>Ongoing infection	Warning and/or error when values are not possible	During technical validation and verification consistency in data entry was evaluated. The most important factors (i.e. ill appearance, indrawings, vital parameters and level of CRP) were 100% uniform.
Algorithm contains different definitions of variables	Calculation performed wrongly	Over- or underestimation of risk	Side effects of antibiotics<br><br>Ongoing infection	Alternative options did not produce significant differences	Observed differences in scores were related to manual faults and different definitions for tachycardia and tachypnea in the technical validation. Risk mitigation measures were applied for these identified critical variables, although no significant differences were observed
Entered data is accessed by unauthorized people	Data leak	Data leak	Immaterial damage	Information security assessment performed	Two-factor authentication is functioning, as evaluated during technical validation and verification.

***Supplementary Table 4**: claims for conformity assessment*

The following claims are made:
• Use of the FKT results in a reduction of antibiotic prescriptions in children who do not profit from antibiotic treatment.
• Use of the FKT results in a reduction of therapeutic failure in children who benefit from antibiotic treatment.
• The FKT application is user-friendly, reflected by a high System Usability Scale / Ottawa Acceptability of Decision Rules Instrument.
• The FKT application is feasible to fill in, so it fits the working environment of an Emergency Department and is not too time-consuming.
• Use of the FKT does not result in an increase of complications.

Supplementary Figure 1: market scan

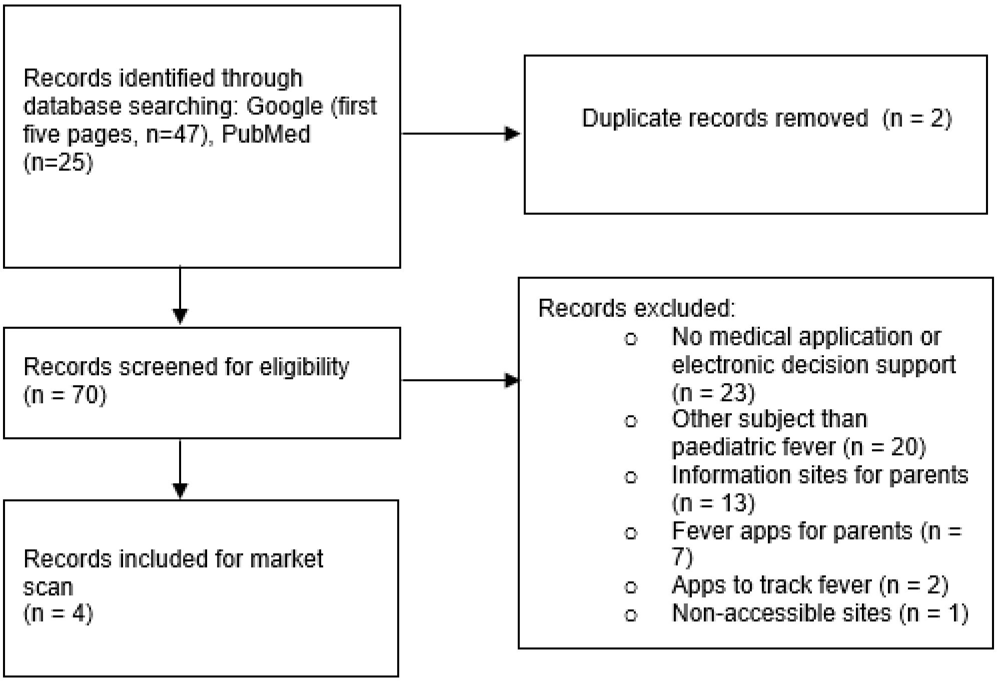

Supplementary Figure 2:

2a: Entry screen of final prototype of Feverkidstool application, with an overview of parameters to enter.

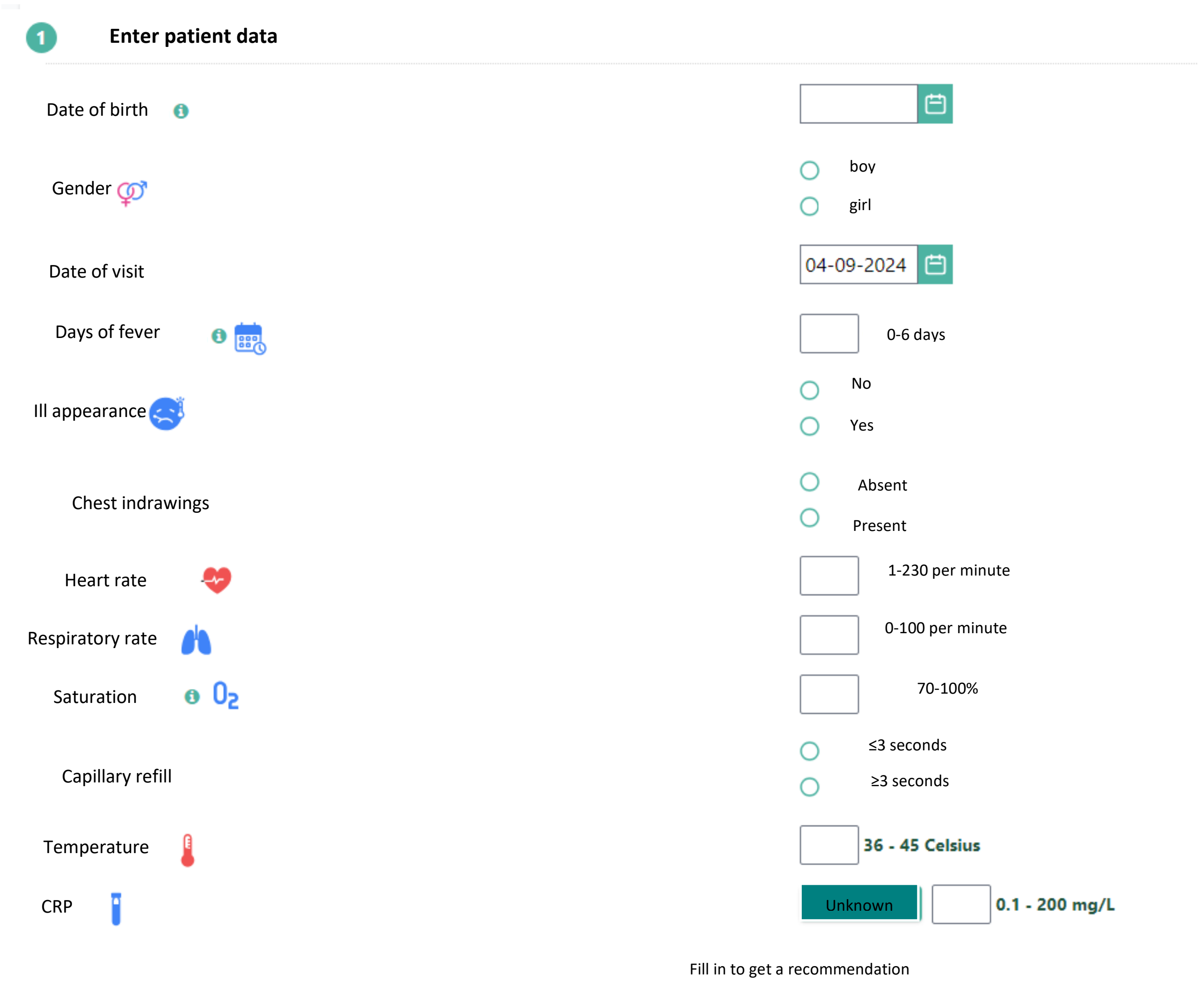

Enter patient data

Date of birth	
Gender	boy / girl
Date of visit	04-09-2024
Days of fever	0-6 days
Ill appearance	No / Yes
Chest indrawings	Absent / Present
Heart rate	1-230 per minute
Respiratory rate	0-100 per minute
Saturation	70-100%
Capillary refill	≤3 seconds / ≥3 seconds
Temperature	36 - 45 Celsius
CRP	Unknown / 0.1 - 200 mg/L

Fill in to get a recommendation

2b: Output screen of final prototype of Feverkidstool application, with a risk calculation provided for bacterial pneumonia and severe bacterial infection. An example of an output is provided. As seen, there is a low risk for bacterial pneumonia and high risk for severe bacterial infection.

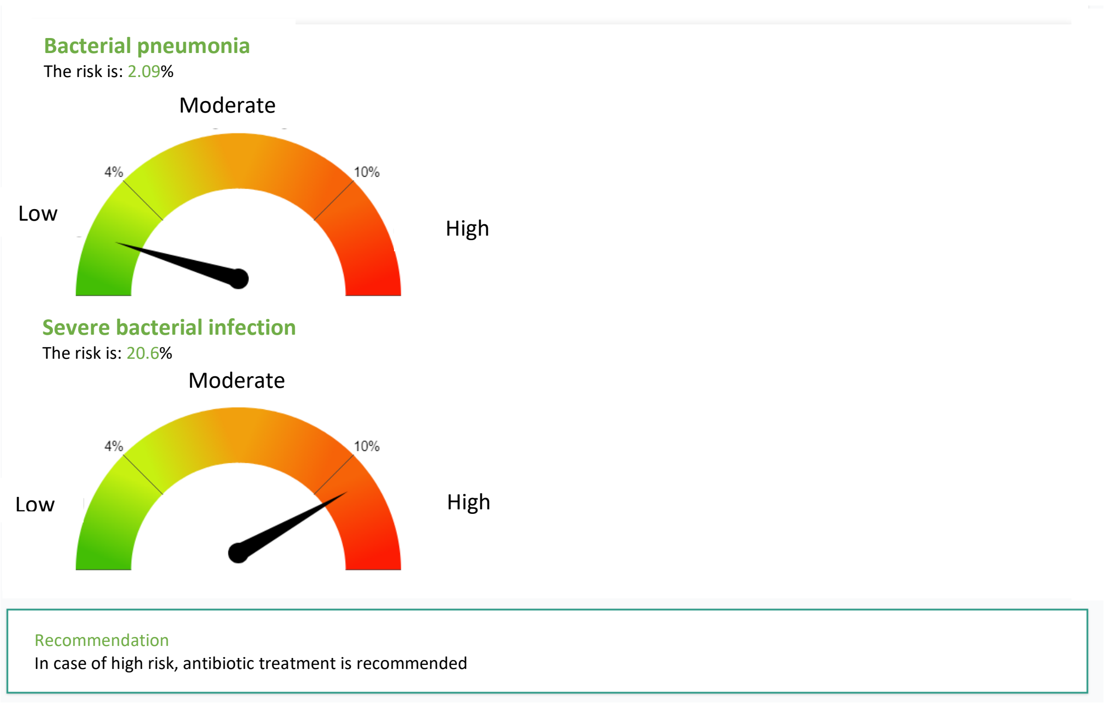

Supplement: Supplementary Tables and Figures [file mmc1.pdf]
